# Confidence, attitudes, beliefs and determinants of implementation behaviours among physiotherapists towards clinical management of low back pain before and after implementation of the BetterBack model of care

**DOI:** 10.1186/s12913-020-05197-3

**Published:** 2020-05-19

**Authors:** Karin Schröder, Birgitta Öberg, Paul Enthoven, Alice Kongsted, Allan Abbott

**Affiliations:** 1 Department of Health, Medicine and Caring Sciences, Division of Prevention, Rehabilitation and Community Medicine, Unit of Physiotherapy, Linköping University, 581 83 Linköping, Sweden; 2 Department of Sports Science and Clinical Biomechanics, University of Southern Denmark, Campusvej 55, 5230 Odense M, Denmark; 3 Nordic Institute of Chiropractic and Clinical Biomechanics, Campusvej 55, 5230 Odense, Denmark

**Keywords:** Clinical guidelines, Physiotherapy, Low back pain, Implementation

## Abstract

**Background:**

Implementing clinical guidelines is challenging. To facilitate uptake, we developed a model of care (BetterBack Model of Care) and an implementation strategy to support management of low back pain in primary care. The aim of this study was to evaluate physiotherapists´ confidence, attitudes and beliefs in managing patients with low back pain before and after a multifaceted implementation of the BetterBack Model of Care. A further aim was to evaluate determinants of implementation behaviours among physiotherapists.

**Methods:**

This clinical trial was an experimental before and after study within a hybrid type 2 effectiveness-implementation trial. The primary outcome was Practitioner Self-Confidence Scale (PCS), secondary outcomes were the Pain Attitude and Beliefs Scale for Physiotherapists (PABS-PT) and Determinants of Implementation Behaviour Questionnaire (DIBQ). Data was analysed using repeated measures ANOVA and pairwise comparisons.

**Results:**

One hundred sixteen physiotherapists answered a questionnaire before, directly after, as well as 3 and 12 months after implementation of the Model of Care. PCS improved over time with a large effect size post implementation (*η*_*p*_^*2*^ = 0.197, *p* < 0.001). Changes in PABS-PT were only significant after 12 months with higher biopsychosocial orientation, (*η*_*p*_^*2*^ = 0.071, *p* < 0.01) and lower biomedical orientation, (*η*_*p*_^*2*^ = 0.136, *p* < 0.001). Directly after the workshop, after 3 and 12 months, physiotherapists had high ratings on all DIBQ domains, (scores > 50) implying that all were potential facilitators of the implementation. However, after 3 months, all domains had significantly decreased except for organisation, social influence and patient expectation domains. However, after 12 months, organisation and social influence domains had significantly decreased while domains such as knowledge, skills and beliefs about capabilities returned to initial levels.

**Conclusions:**

Physiotherapists´ confidence and biopsychosocial orientation increased after implementation and may have the potential to improve management of low back pain in primary care. The implementation behaviour showed mostly facilitating patterns but changed over time, pinpointing a need to repeatedly monitor these changes. This can inform the need for changes of implementation efforts in different phases and support sustainability strategies.

**Trail registration:**

ClinicalTrials.gov NCT03147300 3 May 2017, prospectivly registered.

## Background

Low back pain (LBP) is the leading cause of disability globally and one of the most common causes of visiting primary care [1]. Internationally, there is discordance between evidence and practice concerning the overuse of imaging, spinal injections, passive therapies and surgery and underuse of recommended management [2]. There are several evidence-based clinical guidelines for the management of patients with LBP aiming at different health care practitioners (HCPs) [3]. In Sweden, a previous study by our research group has suggested that the health care process for patients with LBP tends to be fragmented and that only a third of patients on sick leave for musculoskeletal disorders receive evidence-based interventions in primary care [4]. Furthermore, there are still interventions that physiotherapists (PTs) in primary care consider to be relevant despite lack of evidence or treatments effects [5]. Evidence-based physiotherapy interventions are often underused before decision making about spinal surgery. For example, only 58% of the patients on surgical waiting lists have seen a physiotherapist within 12 months prior to a spine surgeon consultation [6]. Guidelines recommend diagnostic triage and non-invasive management including advice to stay active, education on back pain, physical exercise and psychological therapies to empower patients to self-manage their back pain [7–10]. Evidence-based clinical guidelines that are locally adapted and delivered in a model of care (MoC) can potentially bridge the gap between research and practice and facilitate uptake of research findings [11, 12].

In Sweden, direct access and first line treatment by PTs is common. This patient management has been found to be cost effective, safe and well received by patients [13, 14]. However the choice of treatment often reflects clinicians attitudes and beliefs [15]. Recent guidelines for LBP treatment highlight a shift towards a biopsychosocial management approach but the literature shows that PTs are hesitant to address psychosocial factors in LBP and prefer dealing with the more mechanical aspects [16–18]. Using a biomedical orientation has been found to be associated with poor adherence to guidelines as well as advice to delay return to work and activity for patients with LBP [19, 20]. There is therefore a strong need for improved care of patients with LBP in primary care. Self-efficacy and self-confidence are often used interchangeably and Bandura [21] states that high self-efficacy is tied to performance effectiveness in a wide range of situations. An improvement of HCPs´ confidence in managing patients with LBP would probably be tied to a more effective management. Furthermore, there is a need to change clinical behaviour, but guideline implementation is challenging due to lack of evidence regarding the most effective implementation strategy [3]. Implementation strategies for repeated actions to stimulate sustainability and changing HCPs´ behaviour are suggested to be of importance for implementation success [22]. This highlights the importance of evaluating HCPs´ determinants of implementation behaviour over time [23]. The aim of this study was therefore to evaluate physiotherapists´ confidence, attitudes and beliefs in managing patients with LBP before and after a multifaceted implementation of the BetterBack☺ MoC. A further aim was to describe and evaluate determinants of implementation behaviours among physiotherapists. Our hypothesis was that physiotherapist reported confidence would improve while attitudes and beliefs would change towards a more biopsychosocial orientation in managing patients with LBP.

## Method

### Study design

This clinical trial was an experimental before and after study within a hybrid type 2 effectiveness-implementation trial. This hybrid design allows a dual testing of clinical and implementation interventions [24]. The BetterBack☺ MoC was developed based upon two recent clinical guidelines for LBP; from the Danish Health and Medicine Authorities; and the English National Institute for Health and Excellence [8, 9, 25]. To support the development, implementation and evaluation of the MoC an international framework and the Behaviour Change Wheel [26–29] were used. The study protocol was prospectively registered and published with open access, this to increase transparency and avoid reporting bias [30]. The reporting of this study follows the StaRi checklist for implementation studies with an additional intervention description following the TIDieR checklist [31, 32].

### Participants and setting

Eligible participants were registered PTs regularly working with patients with LBP in all 15 primary care rehabilitation clinics in the Östergötland public health care region of Sweden. These 15 clinics formed three rehabilitation units based on municipal and geographical area and organisational structure in Östergötlands health care region. To be included, PTs had to attend a mandatory 2-day BetterBack☺ MoC workshop between March 2017 January 2018. Longitudinal follow-ups were done both by sending out questionnaires and by collecting questionnaires during outreach visits.

### Development and implementation of the MoC

Detailed information about how the content of BetterBack☺ MoC was developed and how the implementation strategy was planned can be found in the protocol [30]. A multifaceted implementation strategy targeting different organisational levels was used [33, 34]. The implementation started with a top-down strategy with a request from the rehabilitation managers [35]. Then a bottom-up strategy with clinicians involved in developing the implementation strategies, locally adapting the clinical guidelines and developing the MoC was used. An existing implementation forum infrastructure was used including managers of the three rehabilitation units and the clinical researchers, with the aim to facilitate the implementation process in the different phases regarding goals, timeline and logistics [36]. During 2016, six trusted clinicians with special skills in treatment of LBP “clinical champions” were selected from each unit by the managers and the clinical researchers (AA, KS) to form a MoC support team [33, 37]. This team adapted two international clinical guidelines to a Swedish context using the Swedish National Board of Health and Welfare methods for guidelines construction [38]. A model of care named “BetterBack☺” was developed by the MoC support team in collaboration with a Danish research group who developed a similar care package [39]. The final recommendation document was expert evaluated by a spinal surgeon and was also presented to the implementation forum.

The following is a description of the BetterBack☺ MoC content structured according to the TIDieR template for intervention description and replication [31].

- WHY: To improve HCP confidence and biopsychosocial orientation in treating LBP through adoption of the BetterBack☺ MoC.
- WHAT: This would require the contents of the MoC to change impeding barrier behaviours such as: 1) Low confidence in skills/capabilities for LBP patient management; 2) Use of a biomedical treatment orientation rather than a biopsychosocial orientation; 3) Low awareness of the model; 4) Beliefs of negative consequences of the model.
- HOW: BetterBack☺ MoC content that differs from routine practice and is used to overcome the modifiable barriers are the following support tools: evidence based guideline recommendations; patient-centred coordinated care pathway; structured subjective and objective assessment proformas; STarT Back Tool [40]; clinical reasoning and process evaluation tool; standardised patient education brochure and material supporting group-based patient education; standardised tools supporting the design and progression of individualised home-based and/or group-based exercise program; web-based educational module and chat forum for PTs.
- WHEN/HOW MUCH/TAILORING: Intervention delivery and dosing is stratified based on the PTs` clinical reasoning regarding risk of pain persistence and progression towards individualised goals.
- PROCEDURE: Procedural descriptions for delivering the BetterBack☺ MoC are included in the following multifaceted implementation strategy. The MoC support team designed and implemented a 2-days workshop (13½ h) for the PTs. This was initiated between March 2017 and January 2018 with 12 to 22 PTs on each occasion in their own clinics. The workshop learning goals and learning activities were based on the Behaviour change wheel [26] and the Behaviour change technique taxonomy [41] (Table 1). To support sustainability, the clinical champions were involved in the education of the clinicians in their clinics, provided reminders during the study, educated new staff and were the local clinical contact person throughout the study. Furthermore, feedback from the MoC support team was delivered to clinics and to the implementation forum during the study to support sustainability. The implementation process was introduced and followed up with one to two outreach visits to the participating clinics by the researchers.

**Table 1:** Linkage between learning goals, activities, behaviour change techniques and mechanism of action for Better Back☺ MoC

**Target behaviour:** Improved HCP confidence and biopsychosocial orientation in treating LBP through adoption of BetterBack☺ MoC				
**Rational based on modifiable barriers to be addressed:** 1. Low confidence in skills/capabilities for improving LBP patient management; 2. Use of a biomedical treatment orientation rather than a biopsychosocial orientation; 3. Low awareness of the model; 4. Beliefs of negative consequences of the model.				
**Strategy to attain target behaviour:** Multifaceted implementation of MoC content to overcome modifiable barriers			**Mechanism of action**	
**Learning goals**	**Learning Activities** **(Intervention functions)**^a^	**Behavioural change techniques used and taxonomy code**	**TDF domains**	**COM-B model**
1) PTs understand evidence-based guideline recommendations for treatment of LBP. 2) PTs understand the theoretical content and clinical benefits of adopting the BetterBack MoC	• A ‘state-of-the-art’ lecture and web-based resources including an overview of the content of evidence-based guideline recommendations (Ed, P, E)	4.1 Instruction on how to perform the behaviour 6.3 Information about other’s approval 9.1 Credible source 9.2 Pros and cons 9.3 Comparative imagining of future outcomes	**Knowledge**	**CAPABILITY DOMAIN**
PTs have the skills to practically use the MoC support tools to: 3) Assist clinical reasoning for matching assessment findings with appropriate diagnosis and stratified treatment 4) Deliver the patient education interventions 5) Deliver exercise interventions	• Demonstration of how to use the MoC support tools (Ed, T, En, M) • Case based practical skills training and role play in small groups using MoC support tools (Ed, T, En) • Peer discussion and reflections upon how they can practically apply the MoC support tools in clinical practice (T, En, M)	1.2 Problem solving 2.2 Feedback on behaviour 3.1 Social support 4.1 Instruction on how to perform the behaviour 6.1 Demonstration of behaviour 6.3 Information about other’s approval 8.1 Behavioural practice/rehearsal 8.7 Graded task 13.2 Framing/re-framing 15.1 Verbal persuasion about capability	**Skills**	
6) PTs have a plan how to start and maintain use of the MoC	• Clinical champion presents an administrative action plan (designed earlier in consensus with clinical colleagues) for the implementation of the MoC at their clinic (Ed, En) • Web-based chat forum for question and feedback (Ed, En)	1.4 Action planning 4.1 Instruction on how to perform the behaviour 12.5 Adding objects to the environment	**Behavioural regulation**	
7) PTs know that their workplace supports delivering the MoC	• Outreached visits before and during the study with managers and clinical champions involved (E, Ed, En)	3.1 Social support 6.3 Information about others’ approval	**Organisation**	**OPPORTUNITY DOMAIN**
8) PTs share knowledge and work together and know whom to ask when they experience difficulty in delivering the MoC	• PTs working together with colleagues in small groups addressing the different parts of the MoC with involvement of the clinical champion (T, M, En)	3.1 Social support 13.1 Identification of self as role model 13.2 Framing/reframing	**Social Influences**	
9) PTs believe that the MoC is appropriate for and accepted by the patient	• A ‘state-of-the-art’ lecture and web-based resources including an overview of the content of evidence-based guideline recommendations (Ed, P, E)	9.3 Comparative imagining of future outcomes	**Patients**	
10) PTs experience that they can tailor the MoC to the patient’s need and clinical practice	• Case based practical skills training working with different patient profiles to address use and tailoring of different components of the MoC (Ed, T, En)	12.1. Restructuring the physical environment 12.2. Restructuring the social environment 12.5 Adding objects to the environment	**Innovation**	
11) PTs feel confident that they can deliver the MoC	• A ‘state-of-the-art’ lecture and web-based resources including an overview of the content of evidence-based guideline recommendations (Ed, P, E) • Demonstration of how to use the MoC support tools (Ed, T, En, M) • Case based practical skills training and role play in small groups using MoC support tools (Ed, T, En) • Peer discussion and reflections upon how they can practically apply the MoC support tools in clinical practice (T, En, M)	1.2 Problem solving 2.2 Feedback on behaviour 3.1 Social support 4.1 Instruction on how to perform the behaviour 6.1 Demonstration of behaviour 6.3 Information about other’s approval 8.1 Behavioural practice/rehearsal 8.7 Graded task 9.1 Credible source 9.2 Pros and cons 9.3 Comparative imagining of future outcomes 13.2 Framing/re-framing 15.1 Verbal persuasion about capability	**Beliefs about Capabilities**	**MOTIVATION DOMAIN**
12) PTs have positive beliefs about the consequences of adopting the MoC	• Presentation of the benefits of using the MoC support tools for assessment, diagnosis and treatment intervention (Ed, P) • Participants discussed the important future outcomes of the MoC implementation based on: 1. A professional perspective; 2. A patient perspective (M)	4.1 Instruction on how to perform the behaviour 5.3 Information about social and environmental consequences 6.3 Information about other’s approval 9.1 Credible source 9.3 Comparative imagining of future outcomes	**Beliefs about consequences**	
13) PTs intend to use the MoC in their clinics in the future	• Facilitated group discussion about practical organisation of delivery the MoC with examples of solutions with clinical champions involved. (P, En)	3.1 Social support 4.1 Instruction on how to perform the behaviour 9.1 Credible source 9.3 Comparative imagining of future outcomes	**Intentions**	

^a^*Ed* Education – Increasing knowledge and understanding, *P* Persuasion – Inducing feelings to stimulate action, *T* Training – Imparting skills, *En* Enablement –Reducing barriers to increase capability,*M* Modelling – Exemplifying to aspire or imitate, *E* Environmental restructuring – changing context (physical/social), *DIBQ* Determinants of Implementation Behaviour Questionnaire, *HCP* Health Care Practitioner, *LBP* Low back pain, *MoC* Model of Care, *PT* Physiotherapist, *COM-B model,* “Capability”, “Opportunity”, “Motivation” and “Behavior” Model

### Variables

**Fig. 1:**
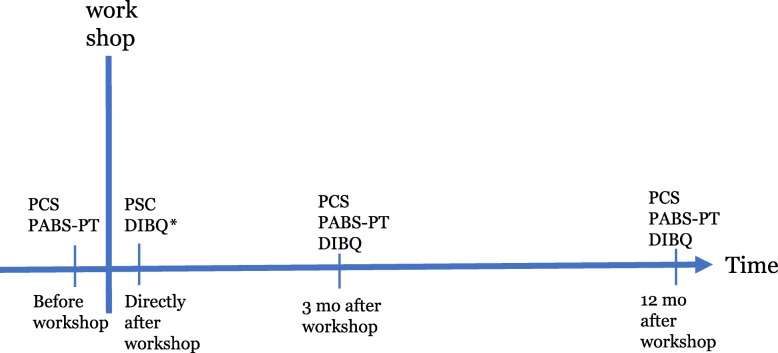
Measure time points. Abbreviations: *PCS* Practitioner Self-Confidence Scale, *PABS-PT* Pain Attitudes and Beliefs Scale for Physiotherapists, *DIBQ* Determinants of Implementation behaviour, *mo* months, * *Expected determinants:* questions were rephrased to expected implementation behaviours since experiences at this time phase was lacking

The participants answered questionnaires before, directly after as well as 3 and 12 months after the workshop (Fig. 1).

#### Implementation outcomes

The primary outcome for the implementation process at the clinician level was the Practitioner Self-Confidence Scale (PCS) mean change from before to 3 months after the workshop [42]. The PCS contains four items with a total score of 20 where 4 represents greatest self-confidence and 20 represents the lowest self-confidence towards clinical management of patients with LBP. Secondary outcomes were PCS mean change from before to directly after and 12 months after the workshop. Another secondary outcome was the 19-item Pain Attitudes and Beliefs Scale for Physiotherapists (PABS-PT) mean change from before to 3 and 12 months after the workshop [43, 44]. PABS-PT consists of two domains, where higher score on the biomedical domain represents higher biomedical orientation PABS-BM (score 10–60). A higher score on the biopsychosocial domain represents higher biopsychosocial orientation PABS-BPS (score 9–54). The PABS-PT is the most commonly used and most thoroughly tested measure of attitudes and beliefs in physiotherapy research [45]. There is evidence for content and construct validity and the PABS-PT has been shown to be associated with several measures of similar constructs such as in the Tampa Scale for Kinesiophobia for health care providers [46].

#### Implementation determinants

**Fig. 2:**
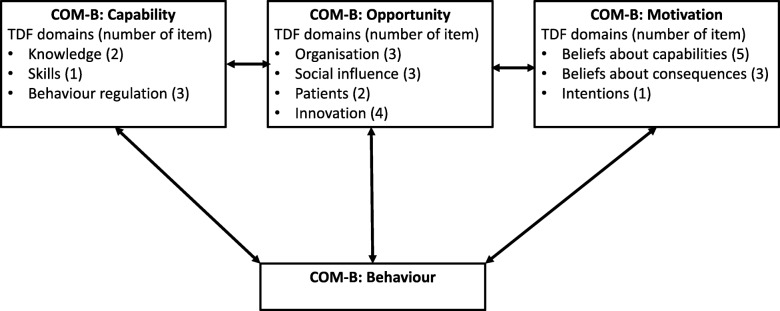
TDF domain linkage to the COM-B model. Abbreviations: *TDF* Theoretical Domain framework, *COM-B* “Capability” "Opportunity" Motivation" and “Behaviour”

The determinants of PTs' behaviour during the implementation of the BetterBack☺ MoC were monitored directly after the workshop as well as after 3 and 12 months using the Determinants of Implementation Behaviour Questionnaire (DIBQ). The questionnaire was originally constructed based on the 18 domains of the Theoretical Domains Framework (TDF) [47] covering possible HCPs' behaviour that can facilitate or hinder the implementation process. The original DIBQ consisted of 93 items but for this to be feasible for use in the current study context, this original DIBQ needed to be reduced and tailored. This was done by using mixed methods in four phases; 1) translation of the original DIBQ into Swedish and Danish; 2) face and content validity assessment by the project team and an expert panel to reduce the number of items and domains. This resulted in a tailored version with 28 items that was tested for 3) feasibility; and 4) construct validity using confirmatory factor analyse (CFA) with regards to item linkage to selected TDF domains. To obtain a satisfactory over all fit, the DIBQ was shortened to the current version with 26 items (Inge Ris, submitted for publication, Dec 2019). The DIBQ can be mapped on to the broader domains of the Capability – Opportunity – Motivation (COM-B system) [48] (Fig. 2). Directly after the 2-day workshop, the DIBQ question items were phrased to report “expected” implementation behaviour since the PTs had not yet used the MoC. The DIBQ at 3 and 12 months retained original wording asking about experiences to investigate the volition phase after use of the BetterBack☺ MoC. The response scale used for each of the 26 items in the DIBQ consists of a 5-point bipolar Likert scale from 1 = ‘strongly agree’, 2 = ‘agree’, 3 = ‘neutral’, 4 = ‘disagree’ 5 = ‘strongly disagree’. The total score was reversed and transformed to a percentage score (0–100), 0 = ‘strongly disagree’, 25 = ‘disagree’, 50 = ‘neutral’, 75 = ‘agree’, 100 = ‘strongly agree’. We suggest that a determinant with a score over 50 can be interpreted as a facilitator of the implementation process. A score between 50 and 75 can be regarded as a weak-moderate facilitator and 75–100 a moderate-high facilitator of the implementation process. Furthermore, a score between 50 and 25 can be interpreted as a weak-moderate barrier and 25–0 a moderate-high barrier of implementation.

### Statistical methods

The PTs' baseline characteristics are presented as means with standard deviations (SD) and proportions (%). All PTs' outcomes were then analysed as differences between before or after workshop and 3 as well as 12 months after workshop (Fig. 1) with Repeated Measures Analysis of Variance (RMANOVA) pairwise comparisons. Main effect sizes as well as pairwise comparisons effect sizes were interpreted with partial eta square (*η*_*p*_^*2*^), where *η*_*p*_^*2*^ = 0*.*01 is considered a small, *η*_*p*_^*2*^ = 0.06 is a medium and *η*_*p*_^*2*^ = 0.14 a large effect size [49]. Missing values were replaced through multiple imputation, based on group data from baseline and the actual time point. Multiple imputation by chained equations procedure (fully conditional specification method in SPSS) with 10 data set was used [50]. Constraints were applied for each variable according to the range of the scale. The mean of 10 imputation set was used for each scale and domains for the different measures. A sensitivity analysis comparing per protocol data without imputation with intention to treat imputed data showed no substantial differences [51]. Statistical analysis was performed using SPSS statistical software for windows (SPSS V25, IBM Corporation, New York, USA). The level of significance was 0.05.

### Study size

Considering a statistical significance of *p* = 0.05 for improvement in PCS directly after the workshop as well as after 3 and 12 months compared to baseline, a correlation between repeated measures = 0.5, an 80% statistical power, a 20% loss of follow-up, a sample of *N* = 43 was needed for a RMANOVA assuming a-priori at least a small Cohens effect size (d = 0.20) based on previous literature described in our study protocol [30]. An internal pilot analysis supported our a-priori sample size calculation [30]. A similar calculation for PABS-PT and DIBQ with their baseline and 2 follow-up time points required a sample of *N* = 50. RMANOVA pairwise comparisons with Bonferroni correction would require a sample size of at least *N* = 73.

## Results

**Fig. 3:**
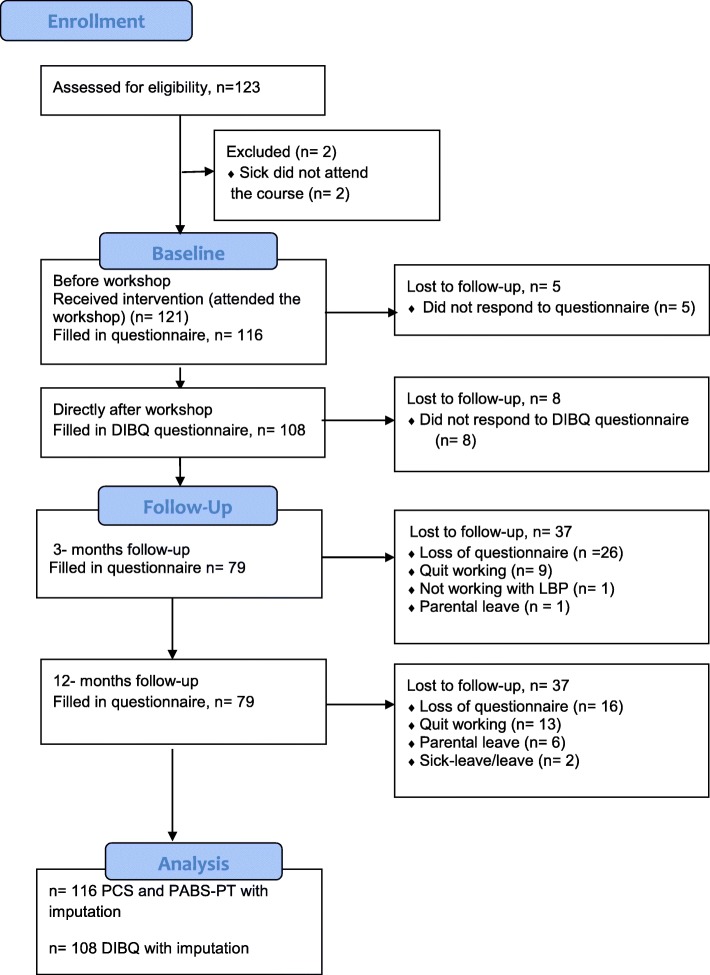
CONSORT flowchart

Figure 3 presents the CONSORT flowchart describing the result of recruitment and data collection. A total of 123 PTs were potential participants for the BetterBack☺ education workshop. Two PTs were absent due to sickness and five PTs declined filling out some of the questionnaires resulting in 116 PTs that completed all the questionnaires before the workshop. Seventy percent were females and the mean age was 38 years. The group with 1–5 years of clinical experience were most common (46%) as well as a bachelor’s degree as the highest education level (89%) (Table 2). There were no significant differences in baseline characteristics between participants and dropouts but there was a slightly higher proportion of clinical years of experience in the dropout group.

**Table 2:** Baseline characteristics of participating physiotherapists

	Total *N* = 121
Age, mean (SD)	38 (12)
Sex: female n (%)	82 (70)
Clinical experience, years, n (%)	
1–5	53 (46)
6–10	21 (18)
11–15	10 (9)
16–20	9 (8)
21–25	8 (7)
> 25	15 (13)
Education level n (%)	
Bachelor’s degree	100 (89)
Post graduate major	8 (7)
Clinical specialist	4 (3)
Post graduate master	3 (23)

Education levels with European Tertiary Credit system

*Abbreviations*: *SD* Standard deviation, *n* number of observations, *PhD* Doctor of Philosophy

The RMANOVA displayed significant improvement in PCS over time with effect size *η*_*p*_^*2*^ = 0.197. Pairwise comparisons directly after as well as 3- and 12-months post workshop (*n* = 116) showed that the PCS was significantly improved compared to before workshop, effect size *η*_*p*_^*2*^ = 0.344, 0.277 and 0.454 respectively (*p* < 0.001) (Table 3). The RMANOVA displayed significant change in PABS-PT over time. Pairwise comparisons between before and 3 months after workshop showed no significant changes in the two subscales of the PABS-PT. Furthermore at 12 months post workshop the PABS-PT biomedical domain had significantly decreased, effect size *η*_*p*_^*2*^ = 0.136, (*p* < 0.001), while the PABS-PT biopsychosocial domain had significantly increased 12 months post workshop effect size *η*_*p*_^*2*^ = 0.071, (*p* < 0.01).

**Table 3:** Changes in physiotherapists’ self-confidence, pain attitudes and beliefs from before to after the workshop (*n* = 116)

	Mean (SD)	Within-subjects effects	Within-subjects simple contrasts	
		*F*-value; *p*-value; Effect Size	Change from baseline Mean (95% CI)	*F*-value; *p*-value; Effect Size
**PCS**		F(2.7, 312) = 28.3; *p* < 0.001; *η*_*p*_^*2*^ = 0.197		
Before	10.4 (2.4)			
Directly after	8.8 (2.1)		−1.6 (− 1.9 to − 1.2)	F(1, 115) = 60.3; *p* < 0.001; *η*_*p*_^*2*^ = 0.344
3 months post	8.9 (2.2)		− 1.5 (− 1.9 to − 1.0)	F(1, 115) = 44.1; *p* < 0.001; *η*_*p*_^*2*^ = 0.277
12 months post	8.7 (2.2)		−1.7 (−2.0 to − 1.3)	F(1, 115) = 95.6; *p* < 0.001; *η*_*p*_^*2*^ = 0.454
**PABS-PT, BM**		F(2, 230) = 7.2; *p* < 0.001; *η*_*p*_^*2*^ = 0.059		
Before	32.0 (7.0)			
3 months post	31.2 (6.9)		−0.8 (−1.8 to 0.1)	F(1, 115) = 2.9; p = 0.09; *η*_*p*_^*2*^ = 0.024
12 months post	30.3 (6.5)		−1.7 (− 2.5 to − 0.9)	F(1, 115) = 18.1; *p* < 0.001; *η*_*p*_^*2*^ = 0.136
**PABS-PT, BPS**		F(2, 230) = 4.2; *p* = 0.016; *η*_*p*_^*2*^ = 0.035		
Before	38.9 (4.8)			
3 months post	39.6 (4.1)		0.7 (− 0.1 to 1.5)	F(1, 115) = 2.9; *p* = 0.09; *η*_*p*_^*2*^ = 0.025
12 months post	40.0 (3.7)		1.1 (0.4 to 1.8)	F(1, 115) = 8.8; *p* < 0.01; *η*_*p*_^*2*^ = 0.071

*Abbreviations*: *SD* Standard Deviation, *CI* Confidence Interval, *η*_*p*_^*2*^ Partial Eta Squared, *PCS* Practitioner Self-Confidence Scale (score 4–20, lower score indicates higher self-confidence), *PABS-PT* Pain Attitudes and Beliefs Scale for Physiotherapists, *BM* Biomedical orientation (score 10–60 indicates higher score higher orientation), *BPS* Biopsychosocial orientation (score 9–54, indicates higher score higher orientation)

**Fig. 4:**
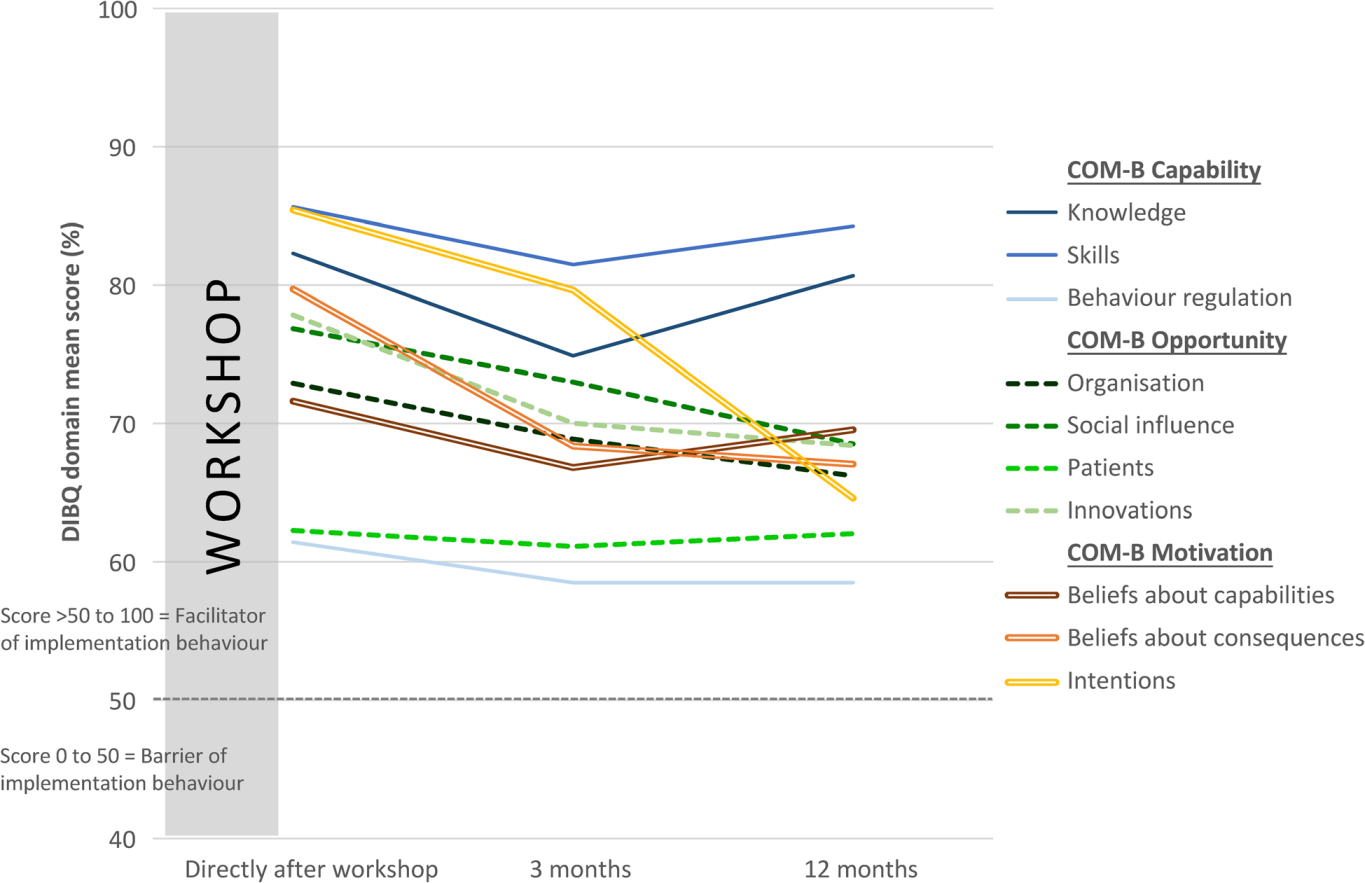
Changes in physiotherapists' determinants of implementation behaviour from baseline (directly after workshop) (*n* = 108). Abbreviations: *COM-B* “Capability” "Opportunity" Motivation" and “Behaviour”, *DIBQ* Determinants of Implementation Behaviour Questionnaire

The RMANOVA displayed high implementation behaviours in all the DIBQ domains over time (scores > 50) implying that all were potential facilitators of implementation with varying strengths. Directly after the 2-day workshop, expected determinants of implementations behaviour domains such as behavioural regulation, organisation, patients and beliefs about capabilities were small-moderate (DIBQ between 50 and 75) potential facilitators of the implementation process (Table 4). Furthermore, domains such as knowledge, skills, behaviour regulation, social influence, innovation, beliefs about consequences and intentions were moderate-high (DIBQ > 75) potential facilitators. Pairwise comparisons between directly after the workshop and after 3 months showed that all domains significantly decreased except for organisation, social influence and patients. Furthermore, at 12 months follow-up knowledge, skills and beliefs about capabilities returned to initial levels. The domains for organisation and social influence were maintained after 3 months but decreased up to 12 months (*p* < 0.01). PTs ratings of patient expectations was maintained at 3- and 12-months follow-up (Table 4 and Fig. 4).

**Table 4:** Changes in physiotherapists' determinants of implementation behaviour from baseline (directly after workshop) (*n* = 108)

			Within-subjects effects	Within-subjects simple contrasts	
		Mean (SD)	*F*-value; *p*-value; Effect Size	Change from baseline Mean (95% CI)	*F*-value; *p*-value; Effect Size
**COM-B Capability**	**Knowledge**		F(2, 214) = 12.6; *p* < 0.001; *η*_*p*_^*2*^ = 0.105		
	Directly after	82.3 (14.4)			
	3 months post	74.9 (17.1)		− 7.4 (− 10.8 to − 4.1)	F(1, 107) = 19.1; *p* < 0.001; *η*_*p*_^*2*^ = 0.152
	12 months post	80.7 (16.8)		−1.6 (− 4.7 to 1.5)	F(1, 107) = 1.1; *p* < 0.30; *η*_*p*_^*2*^ = 0.010
	**Skills**		F(1.7, 182) = 2.3; *p* = 0.11; *η*_*p*_^*2*^ = 0.021		
	Directly after	85.6 (18.2)			
	3 months post	81.5 (19.4)		− 4.2 (− 8.3 to − 0.1)	F(1, 107) = 4.1; *p* < 0.05; *η*_*p*_^*2*^ = 0.037
	12 months post	84.3 (19.5)		− 1.4 (− 5.9 to 3.1)	F(1, 107) = 0.4; *p* < 0.54; *η*_*p*_^*2*^ = 0.004
	**Behavioural regulation**		F(2, 214) = 2.1; *p* = 0.13; *η*_*p*_^*2*^ = 0.019		
	Directly after	61.4 (18.5)			
	3 months post	58.5 (17.6)		−2.9 (− 6.4 to 0.5)	F(1, 107) = 2.9; *p* = 0.03; *η*_*p*_^*2*^ = 0.026
	12 months post	58.5 (17.0)		− 2.9 (− 6.4 to 0.6)	F(1, 107) = 2.7; *p* = 0.03; *η*_*p*_^*2*^ = 0.025
**COM-B Opportunity**	**Organisation**		F(1.9, 201) = 5.1; *p* < 0.01; *η*_*p*_^*2*^ = 0.046		
	Directly after	72.9 (23.0)			
	3 months post	68.9 (20.1)		− 4.1 (− 8.2 to 0.1)	F(1, 107) = 3.8; *p* = 0.05; *η*_*p*_^*2*^ = 0.034
	12 months post	66.2 (16.1)		−6.7 (− 11.4 to 2.0)	F(1, 107) = 8.1; *p* < 0.01 *η*_*p*_^*2*^ = 0.070
	**Social influence**		F(2, 214) = 8.6; *p* < 0.001; *η*_*p*_^*2*^ = 0.074		
	Directly after	76.9 (17.9)			
	3 months post	73.0 (20.4)		−3.9 (−8.0 to 0.3)	F(1, 107) = 3.4; *p* = 0.07; *η*_*p*_^*2*^ = 0.031
	12 months post	68.5 (16.2)		− 8.3 (−12.3 to − 4.3)	F(1, 107) = 17.1; *p* < 0.001; *η*_*p*_^*2*^ = 0.138
	**Patients**		F(2, 214) = 0.3; *p* = 0.77; *η*_*p*_^*2*^ = 0.002		
	Directly after	62.3 (14.1)			
	3 months post	61.1 (14.7)		−1.2 (−4.7 to 2.3)	F(1, 107) = 0.4; *p* = 0.51; *η*_*p*_^*2*^ = 0.004
	12 months post	62.0 (13.3)		−0.2 (− 3.7 to 3.2)	F(1, 107) = 0.2; *p* = 0.90; *η*_*p*_^*2*^ < 0.001
	**Innovation**		F(1.9, 203) = 23.4; p < 0.001; *η*_*p*_^*2*^ = 0.180		
	Directly after	77.8 (14.5)			
	3 months post	70.0 (16,5)		−7.8 (−10.9 to −4.8)	F(1, 107) = 25.7; *p* < 0.001; *η*_*p*_^*2*^ = 0.194
	12 months post	68.4 (18.3)		−9.4 (− 12.6 to −6.3)	F(1, 107) = 35.9; *p* < 0.001; *η*_*p*_^*2*^ = 0.251
**COM-B Motivation**	**Beliefs about Capabilities**		F(2, 214) = 2.9; *p* = 0.06; *η*_*p*_^*2*^ = 0.026		
	Directly after	71.6 (16.5)			
	3 months post	66.8 (19.7)		−4.2 (−8.3 to −0.1)	F(1, 107) = 5.7; *p* = 0.02; *η*_*p*_^*2*^ = 0.051
	12 months post	69.5 (17.2)		−2.1 (−6.4 to 2.2)	F(1, 107) = 0.9; *p* = 0.34; *η*_*p*_^*2*^ = 0.009
	**Beliefs about consequences**		F(2, 214) = 41.2; *p* < 0.001; *η*_*p*_^*2*^ = 0.278		
	Directly after	79.7 (15.0)			
	3 months post	68.4 (19.4)		−11.3 (− 14.3 to −8.4)	F(1, 107) = 57.4; *p* < 0.001; *η*_*p*_^*2*^ = 0.349
	12 months post	67.1 (18.7)		−12.7 (− 15.7 to −9,6)	F(1, 107) = 65.8; *p* < 0.001; *η*_*p*_^*2*^ = 0.381
	**Intentions**		F(1.8, 196) = 38.6; *p* < 0.001; *η*_*p*_^*2*^ = 0.265		
	Directly after	85.4 (21,7)			
	3 months post	79.6 (23.5)		−5.8 (−10.0 to − 1.6)	F(1, 107) = 7.4; *p* < 0.01; *η*_*p*_^*2*^ = 0.065
	12 months post	64.6 (25.6)		−20.8 (− 26.3 to − 15.3)	F(1, 107) = 56.9; *p* < 0.001; *η*_*p*_^*2*^ = 0.347

*Abbreviations*: *SD* Standard Deviation, *CI* Confidence Interval, *η*_*p*_^*2*^ Partial Eta Squared, *COM-B* “Capability”, “Opportunity”, “Motivation” and “Behaviour” model

## Discussion

The key findings in this study were improved confidence, change towards a more biopsychosocial orientation and high determinants of implementation behaviours among PTs after implementation of the BetterBack☺ MoC. Our hypothesis that physiotherapists' confidence, attitudes and beliefs in managing patients with LBP improve after a multifaceted implementation of the BetterBack☺ MoC was confirmed. The PTs´ confidence in managing patients with LBP improved already at directly after, 3 as well as at 12 months after the implementation. A study of PTs treating osteoarthritis had used a modified version of PCS and showed similar result after implementing a new physiotherapy training program in managing patients with osteoarthritis [52]. In that study, the improved self-confidence after implementation changed clinical behaviour, but some of these positive changes were lost over time.

A change in attitudes and beliefs towards a more biopsychosocial orientation and less biomedical orientation was found at 3 months but were statistically significant first at 12 months after implementation. The PTs in our study reported a high biopsychosocial orientation from the start, leaving less room for improvement, but despite this a significant increase was still obtained. Several studies have demonstrated that HCPs´ attitudes and beliefs are associated with the treatment recommendations they give to the patients and that this may transfer to a better management of LBP in primary care [19, 53]. A qualitative study evaluated LBP interventions with a biopsychosocial focus found that 13 qualified PTs experienced improved confidence to manage the biopsychosocial dimensions after an average of nine workshops with cognitive functional therapy training [54]. Furthermore, a voluntarily 8-day university course [55] as well as a 7-h PT workshop [56] focusing on biopsychosocial treatment showed a similar result as in the current study. These three studies [54–56] however only performed follow-up directly after the training/course leaving long-term effects unclear. Another limitation is the small cohorts of PTs (12 to 42 PTs) with a selection bias towards motivated participants.

According to the diffusion theory, people in a group vary from “early adopters” to “laggards” who adopt late to innovations [57]. For example, laggards may consider guidelines as a threat to professional autonomy and clinical reasoning, or they may have attitudes and beliefs that are not in line with the guidelines [58]. In the present study we could utilise an existing regional infrastructure (implementation forum) as a top-down strategy to action mandatory MoC education for all PTs in the public health care region. Our rational was to reach all PTs within the organisation to improve guideline adherent care, despite the potential of including both early adopters and laggards. This representative sampling may partly explain why there was no short-term effect but only a long-term effect on PTs´ attitudes and beliefs as this is likely to require longer time and real-world practice. We also chose to include a bottom-up strategy utilising clinical champions, irrespective of their previous attitudes and beliefs, but rather their trustworthiness within the organisation and among colleagues. This allowed for the inclusion of potential critical views in the MoC development process to prepare for and manage such barriers during the implementation phase.

In the initial implementation process, PTs had high expectations for the BetterBack☺ MoC, and all DIBQ domains could have a potential facilitating role on the implementation process. This overall pattern of expected facilitating determinants of implementation behaviour directly after the 2-day workshop could be a result of successful motivation and initiation of the implementation. However, after 3 and 12 months of applying the model (volition stage), the initial expectations decreased in most DIBQ domains but maintained a potential facilitating role. Therefore, PTs initially high expectations may facilitate the implementation process to start with, but the decrease of these expectation during the volition phase underscores a need of sustainability strategies.

With regards to capability related determinants of behavioural change goals “COM-B Capability”, linked DIBQ domains showed a pattern of short-term decrease followed by long-term sustainability. For opportunity related determinants of a behavioural change goal “COM-B Opportunity”, PTs maintained views that patients have positive expectations for the MoC (DIBQ patient domain) both in the short- and long-term. However, the organisation and social influence DIBQ domains showed a pattern of short-term sustainability but decreased in the long term. Furthermore, the innovation DIBQ domain decreased both in the short and long term. Similarly, for motivation related determinants of behavioural change goals “COM-B Motivation”, all DIBQ domains showed a decrease both in the short and long term except for beliefs about capabilities that returned to initial levels after 3 months.

In contrast to the present findings, organisational resources and support are usually described as barriers to implementation efforts [18, 59, 60]. In the present study, clinical managers and clinical champions were likely important facilitators in providing opportunities for positive social support, reinforcement of action plans and motivating colleagues intentions to use the MoC. However, this is likely to require more regular focus over time to maintain a facilitatory effect on motivation and opportunity related determinants. Organisational literature suggests that clinical champions play an important role in communicating and building relationships throughout the organisation [61]. This highlights the importance of both a top-down and a bottom-up involvement during MoC implementation with both clinical managers and carefully selected clinical champions involved in iterative sustainability efforts.

The current implementation strategy showed that the impact of a well-planned guideline development, education and use of clinical champions gave good effect and mostly facilitated the implementation process. The implementation strategy did not fully meet the demands on the organisational level since these aspects, as measured by the DIBQ, showed a decreasing facilitation trend over time. Literature also supports assessing sustainability over multiple time points to capture its possible dynamic nature [62]. The use of DIBQ repeatedly during the implementation can serve as a tool to catch changes of facilitators and barriers. This might be useful to inform and act upon for continuous improvement in an implementation strategy. In the case of this study, if we had immediately analysed and acted upon the DIBQ information, this could have provided more support during the late phase of implementation on an organisational level.

### Strengths and limitations

This is the first study using the DIBQ with longitudinal monitoring. A strength in this study was that it is representative for the health care region, with 123 eligible PTs and 121 educated and 116 analysed. The present study contained publicly financed PTs, however a previous study has compared PTs working privately and those publicly financed and differences in orientations emerged [63]. The study displayed a higher degree of biopsychosocial orientation in publicly financed PTs, indicating that the result of the present study is likely more generalisable in the public health care system. The present study contained different practice sizes from 2 to 3 PTs up to large practices with more than 20 PTs. The results can therefore be generalisable for different practice sizes. Since Östergötland is a representative county for Sweden, the findings of the present study can be generalisable at national level. Results of our study may also be generalisable in similar health care systems internationally.

A possible strength in this study design was the use of a theory driven multifaceted implementation strategy [64]. A recent review about PT-delivered cognitive-behavioural interventions found no study providing intervention description with accessible training materials to allow replication [17]. In our on-line protocol we have all the material accessible [30]. A potential bias was that the researchers handed out the questionnaires which may result in more expected and socially acceptable answers. Further work is needed to analyse what influence the implementation of the BetterBack☺ MoC has on PTs' adherence to clinical guidelines, potential causal mechanisms and how these relate to patient outcomes. Future studies are needed investigating the use of the DIBQ as a tool to identify and immediately act upon barrier behaviours aiding sustainability of implementation over time in dynamic health care systems.

## Conclusions

Physiotherapists' confidence and biopsychosocial orientation increased after implementation of BetterBack☺ MoC and may have the potential to improve management of LBP in primary care. PTs' high expectations measured by determinants of implementation behaviour may facilitate the implementation process to start with, but decreased during the volition phase, underscoring a need of sustainability strategies. The use of repeated screening with the DIBQ can support continuous adaptation towards a more focused implementation strategy.

## Data Availability

The datasets generated during and/or analysed during the current study are not publicly available as participants consent and institutional approval for this activity were not obtained.

## Abbreviations

COM-B: Capability Opportunity Motivation-Behaviour model
DIBQ: Determinants of Implementation Behaviour Questionnaire
HCP: Health Care Practitioner
LBP: Low Back Pain
MoC: Model of Care
PABS-PT: Pain Attitude and Beliefs Scale for Physiotherapists
PABS-BM: Pain Attitude and Beliefs Scale-Biomedical score
PABS-BPS: Pain Attitude and Beliefs Scale-Biopsychosocial score
PCS: Practitioner Confidence Scale
PT: Physiotherapist
TDF: Theoretical Domains Framework
